# Mushroom *Ganoderma lucidum* Prevents Colitis-Associated Carcinogenesis in Mice

**DOI:** 10.1371/journal.pone.0047873

**Published:** 2012-10-30

**Authors:** Daniel Sliva, Jagadish Loganathan, Jiahua Jiang, Andrej Jedinak, John G. Lamb, Colin Terry, Lee Ann Baldridge, Jiri Adamec, George E. Sandusky, Shailesh Dudhgaonkar

**Affiliations:** 1 Cancer Research Laboratory, Methodist Research Institute, Indiana University Health, Indianapolis, Indiana, United States of America; 2 Department of Medicine, Indiana University School of Medicine, Indianapolis, Indiana, United States of America; 3 Department of Pathology, Indiana University School of Medicine, Indianapolis, Indiana, United States of America; 4 Indiana University Cancer Center, Indiana University School of Medicine, Indianapolis, Indiana, United States of America; 5 Department of Pharmacology and Toxicology, University of Utah, Salt Lake City, Utah, United States of America; 6 Bindley Bioscience Center, Purdue University, West Lafayette, Indiana, United States of America; The University of Hong Kong, Hong Kong

## Abstract

**Background:**

Epidemiological studies suggest that mushroom intake is inversely correlated with gastric, gastrointestinal and breast cancers. We have recently demonstrated anticancer and anti-inflammatory activity of triterpene extract isolated from mushroom *Ganoderma lucidum* (GLT). The aim of the present study was to evaluate whether GLT prevents colitis-associated carcinogenesis in mice.

**Methods/Principal Findings:**

Colon carcinogenesis was induced by the food-borne carcinogen (2-Amino-1-methyl-6-phenylimidazol[4,5-*b*]pyridine [PhIP]) and inflammation (dextran sodium sulfate [DSS]) in mice. Mice were treated with 0, 100, 300 and 500 mg GLT/kg of body weight 3 times per week for 4 months. Cell proliferation, expression of cyclin D1 and COX-2 and macrophage infiltration was assessed by immunohistochemistry. The effect of GLT on XRE/AhR, PXR and rPXR was evaluated by the reporter gene assays. Expression of metabolizing enzymes CYP1A2, CYP3A1 and CYP3A4 in colon tissue was determined by immunohistochemistry. GLT treatment significantly suppressed focal hyperplasia, aberrant crypt foci (ACF) formation and tumor formation in mice exposed to PhIP/DSS. The anti-proliferative effects of GLT were further confirmed by the decreased staining with Ki-67 in colon tissues. PhIP/DSS-induced colon inflammation was demonstrated by the significant shortening of the large intestine and macrophage infiltrations, whereas GLT treatment prevented the shortening of colon lengths, and reduced infiltration of macrophages in colon tissue. GLT treatment also significantly down-regulated PhIP/DSS-dependent expression of cyclin D1, COX-2, CYP1A2 and CYP3A4 in colon tissue.

**Conclusions:**

Our data suggest that GLT could be considered as an alternative dietary approach for the prevention of colitis-associated cancer.

## Introduction

The Western diet consists of large amounts of consumed hamburgers, well done steaks, grilled chicken and fish. In spite of the fact that poultry and fish are generally healthier than red meat, during the cooking process, heterocyclic amine (HCA) compounds are formed [1]. One of them, 2-Amino-1-methyl-6-phenylimidazol[4,5-*b*]pyridine (PhIP) is one of the most abundant HCAs in heated meat, poultry and fish. Intake of well-done red meat, containing PhIP, was associated with increased risk of colon adenomas [2], [3]. Inflammation plays a crucial role in the cancer development [4], and chronic inflammation leading to inflammatory bowel disease (Crohn's disease and ulcerative colitis) is associated with colorectal cancer risk [5].

Although the importance of food, nutrition, physical activity in the prevention of cancer has been recently acknowledged [6], some food or nutritional products with healthy potential are largely ignored. Among these overlooked or sparsely used natural products are mushrooms. However, four recent epidemiological studies from Asia demonstrated inverse correlation between mushroom intake and gastric, gastrointestinal and breast cancer, respectively [7]–[10]. The anticancer activities of mushrooms were usually associated with the stimulation of the immune system by polysaccharides, predominantly β-glucans [11]. On the other hand, mushrooms contain minerals, vitamins (e.g. thiamin, riboflavin, ascorbic acid, and vitamin D), amino acids, and other organic compounds [12].

The mushroom *Ganoderma lucidum* was used in the traditional Chinese medicine (TCM) and is commonly used in the forms of tea, and dietary supplements to promote health. The major biologically active components identified in *G. lucidum* are polysaccharides, which stimulate the immune system, and lanostane-type triterpenes, which directly target cancer cells [13]. Animal studies with *G. lucidum* extracts demonstrated that triterpene fractions, containing ganoderic and lucidenic acids, inhibited growth and metastasis of Lewis lung carcinoma and human hepatoma cells in mice [14], [15], whereas a water soluble extract from *G. lucidum* mycelia inhibited formation of ACF and reduced the size of colonic tumors induced by azoxymethane and N,N′-dimethylhydrazine in rats and mice, respectively [16], [17].

In the present study we evaluated *G. lucidum* triterpene extract (GLT) in the animal model of the food-borne carcinogen (PhIP) and inflammation (DSS) induced colon carcinogenesis mice. Here, we show that GLT prevented formation of colonic tumors, inhibited focal hyperplasia and reduced the amount of ACF. Moreover, GLT also prevented colon inflammation and reduced the amount of colon infiltrating macrophages. Finally, we have also shown that GLT significantly down-regulated PhIP/DSS-dependent expression of CYP1A2 and CYP3A4 in colon tissue.

## Results

### *Ganoderma lucidum* triterpene extract (GLT) inhibits colon carcinogenesis

**Figure 1:**
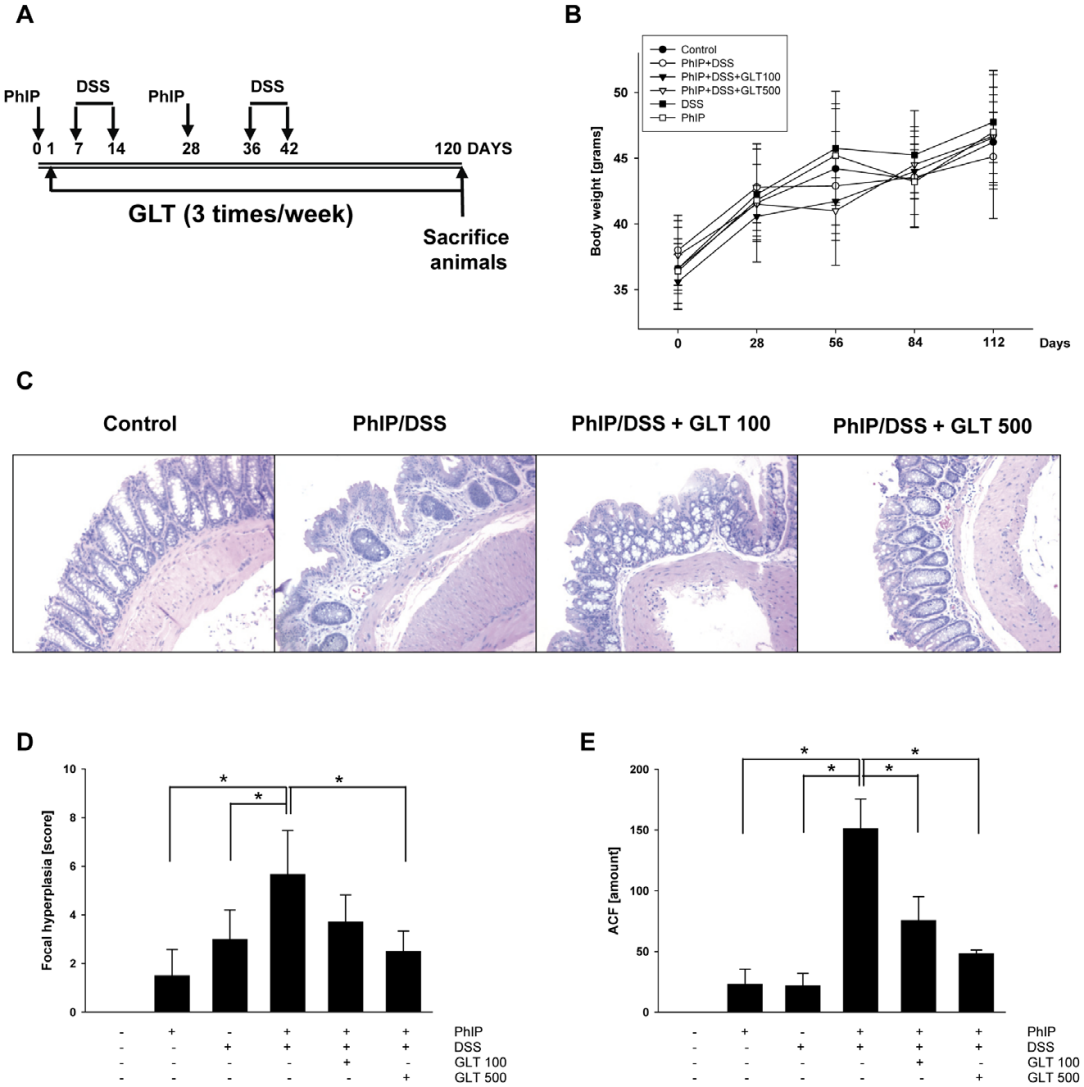
GLT suppresses PhIP/DSS induced formation of colon tumors and inhibits focal hyperplasia and ACF formation. (A) Schematic of the animal treatment. The details of the treatment are described in *Materials and Methods*. (B) Body weight of control animals (black circle), animals treated with PhIP (white square), DSS (black square), PhIP/DSS (white circle), PhIP/DSS+GLT 100 mg/kg of body weight (black triangle), and PhIP/DSS+GLT 500 mg/kg of body weight (white traingle) during the experiment. (C) H&E staining of representative samples from animal experiments described in Figure 1A. (D) Focal hyperplasia was evaluated by the histological analysis after H&E staining in colon tissue samples as described in *Materials and Methods*. Results are means ± SD (n = 6–9 mice/per group). (E) ACF formation was evaluated after methylene blue staining as described in *Materials and Methods*. Results are means ± SD (n = 10 foci/3 mice/per group), *p<0.05 by ANOVA.

In order to evaluate whether GLT suppresses colon carcinogenesis induced by PhIP, we have modified an animal model where the carcinogenic effect of PhIP is further induced by the inflammation with DSS [18]. The mice treated with PhIP, DSS or their combination with GLT (Fig. 1A) did not demonstrate any sign of toxicity as shown by the even increase of body weight among the groups (Fig. 1B). Although our experiments started with 10 animals per group, some of the animals died during the experiment. Thus, we observed slightly increased mortality in the control group (1 dead animal), groups in animals treated with DSS (2 deaths), and PhIP/DSS (1 death), whereas GLT treatment further increased mortality of experimental animals (PhIP/DSS+100 mg GLT/kg of body weight - 3 deaths, PhIP/DSS+500 mg GLT/kg of body weight - 4 deaths). However, this increased mortality was not statistically significant, and the pathological analysis did not show any changes among the dead animals. Although DSS induced slight diarrhea and bloody stool after 5–7 days in mice exposed to 2% DSS in the drinking water, this effect was only transient and all animals produced normal stool during the experiment.

The presence of colonic neoplasm was evaluated macroscopically in all treatment groups and nodular, polypoid, or flat-type colonic tumors were observed in proximal, middle, and distal colon. As seen in Table 1, we were not able to detect any tumors in control or DSS groups and only one tumor in the PhIP group, whereas the combination treatment of PhIP and DSS markedly induced the tumor incidence and tumor multiplicity. However, the treatment with GLT reduced the tumor incidence and the tumor multiplicity at 100 mg GLT/kg of body weight and at 500 mg GLT/kg of body weight, respectively (Table 1). Neoplastic index was calculated by evaluating the severity of neoplastic lesions as described in *Materials and Methods*. Although PhIP itself significantly induced median value for the neoplastic index to 1, this effect was further increased when DSS was added to the drinking water (Table 1). However, GLT treatment only moderately decreased the neoplastic index in the treated animals to 2 at the dose 100 mg GLT/kg and 1.5 at the dose 500 mg GLT/kg, respectively (Table 1).

**Table 1:** Effect of GLT on the PhIP/DSS induced colon carcinogenesis and inflammation.

Treatment	Tumor incidence (animals with tumors)	Tumor multiplicity (tumors per animal)	Neoplastic index	Colon length (cm)
**Control**	0/9 (0%)	0	0 (0,0)	14.1±1.2
**PhIP**	1/10 (10%)	0.1±0.32	1 (0,1)^a^	14.5±1.1
**DSS**	0/8 (0%)	0	0 (1,0)	11.6±1.8^a^
**PhIP/DSS**	9/9 (100%)^a^	5.6±2.01^a^	3 (1,4)^b^	11.2±2.0^a^
**PhIP/DSS+100 GLT**	5/7 (71%)	2.1±2.12	2 (1,3)	13.4±0.8^b^
**PhIP/DSS+500 GLT**	2/6 (33%)^b^	0.7±1.21^b^	1.5 (0,3)	14.6±1.3^b^

Tumor incidence are summarized using percentage of animals with tumors and compared across groups using Fisher's exact test and the Bonferonni correction for multiple comparisons: ^a^ p<0.001 PhIP/DSS vs control, PhIP, DSS; ^b^ p<0.02 PhIP/DSS+500 GLT vs PhIP/DSS.

Tumor multiplicity are summarized using mean ± SD and compared across all group using Kruskal-Wallis one way analysis of variance on ranks and the Dunn's method for the multiple comparisons: ^a^ p<0.05 PhIP/DSS vs control PhIP, DSS; ^b^ p<0.05 PhIP/DSS+500 GLT vs PhIP/DSS.

Neoplastic index is summarized using median (min, max) and compared across groups using the Kruskal-Wallis test. Comparisons of each group to control performed using Mann-Whitney U tests with significance levels adjusted using the Bonferroni correction: ^a^ p<0.001 control vs PhIP; ^b^ p<0.001 control vs PhIP/DSS.

Data for colon length summarized using mean ± SD and compared across all group using ANOVA and Dunnett's post hoc test: ^a^p<0.001 control vs DSS, control vs PhIP/DSS; ^b^p<0.001 PhIP/DSS vs PhIP/DSS+100 GLT, PhIP/DSS vs PhIP/DSS+500 GLT.

### GLT suppresses focal hyperplasia and aberrant crypt foci (ACF) formation

The earliest phases of colorectal oncogenesis occur in the normal mucosa, with a disorder of cell replication and further induction of epithelial hyperplasia [19]. The focal hyperplasia was evaluated in colonic tissues after H&E staining. Histological analysis (Fig. 1C) demonstrated that PhIP and DSS induced hyperplasia of focal colonic crypt, adenomas and adenocarcinomas in the mucosa layer of the colon with some residual parts of normal colonic architecture. GLT treatment (100 mg/kg) partially restored the normal colonic architecture. At the higher concentration of GLT (500 mg/kg), no visible areas of colon adenocarcinomas were detected and only distinct areas of focal colonic epithelial hyperplasia were identified with visible areas of normal colonic architecture. As seen in Figure 1C and D, PhIP (1.5±1.07) and DSS (3.0±1.20) alone only moderately increased colonic focal hyperplasia, whereas the combination of PhIP and DSS significantly (5.7±1.80, p<0.05) induced focal hyperplasia. This PhIP and DSS dependent focal hyperplasia was suppressed by the GLT treatment in a dose response manner at 100 mg GLT/kg (3.7±1.11) and at 500 mg GLT/kg (2.5±0.84, p<0.05) (Fig. 1D). The aberrant crypt foci (ACF) formation is a putative precursor to colorectal adenomas and a potential biomarker for colorectal carcinoma [20]. As recently demonstrated, whereas PhIP itself did not induce the number of ACF, the combination of PhIP and DSS markedly stimulated formation of ACF in male C57BL/6J mice [21], [22]. Interestingly, we observed a slight induction of ACF by PhIP (23±12.5) or DSS (22±9.9) only, whereas the combination of PhIP and DSS significantly (151±24.1, p<0.05) induced formation of ACF (Fig. 1E). Notably, the amount of ACF was suppressed by GLT in a dose response manner at 100 mg GLT/kg (76±19.5, p<0.05) and at 500 mg GLT/kg (48±2.9, p<0.05).

**Figure 2:**
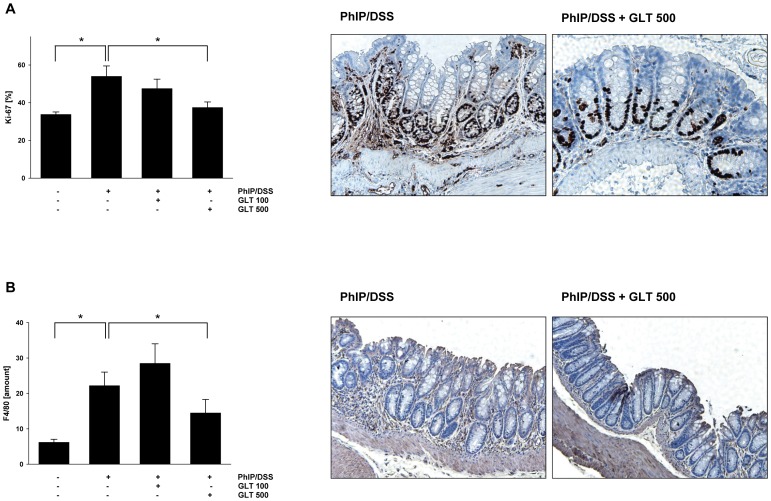
GLT down-regulates expression of Ki-67 and suppresses the amount of infiltrated macrophages. Immunohistochemistry and quantification of (A) Ki-67 and (B) F4/80 positive cells were performed as described in *Materials and Methods*. Results are means ± SD (n = 4–5 mice/per group), *p<0.05 by ANOVA. Representative pictures from (A) Ki-67 and (B) F4/80 staining in the PhIP/DSS group and the PhIP/DSS group treated with GLT (500 mg/kg of body weight) 3 times/week for 120 days.

Increased proliferation of colon epithelial cell, characterized as hyperplasia, can be detected with the Ki-67 proliferation marker [23]. Therefore, we evaluated whether GLT suppresses the amount Ki-67 positive cells in colon tissue in our experimental conditions. As seen in Figure 2A, PhIP and DSS induced the amount of Ki-67 positive cells, whereas the treatment with GLT decreased Ki-67 cells in a dose response manner.

### GLT suppresses colon inflammation

DSS is a widely used inducer of colonic inflammation in experimental models of colitis and previous studies demonstrated that DSS further accelerate colon carcinogenesis induced by PhIP [18], [24]. The major characteristics of DSS induced inflammation/colitis are the weight loss and the shortening of the large intestine in DSS treated animals [25]. In our experiments, repeated applications of 2% DSS in water for one week at days 7–14 and 36–42 (Fig. 1) significantly shortened the colon length in DSS and in PhIP/DSS treated animals, respectively (Table 1). In contrast, GLT treatment prevented the shortening of colon lengths to the levels comparable to the control group, and this effect was dose dependent (Table 1). Nevertheless, DSS alone, combination of PhIP and DSS or the treatment with GLT did not affect the body weight of experimental animals (Fig. 1B).

DSS-dependent colon inflammation is associated with the increased infiltration of macrophages [26]. To evaluate whether the amount of macrophages is increased in the PhIP/DSS induced colon cancer model and whether GLT affects the amount of macrophages in these animals, colon tissues were stained with F4/80 antibodies. As seen in Figure 2B, DSS and PhIP induced infiltration of macrophages was significantly reduced in colon tissue in animals treated with 500 mg GLT/kg.

### GLT is not toxic and prevents colon carcinogenesis and inflammation

Smina et al [27] recently demonstrated that a single dose (5000 mg/kg of body weight) or daily dose for 30 days (500 mg/kg of body weight) of total triterpenes isolated from *Ganoderma lucidum* are not toxic, To confirm that GLT is not toxic, GLT (0, 125, 250 and 500 mg/kg of body weight) was administered orally for 5 days and the effects on the liver, kidney and glucose and lipids levels evaluated after one week. As seen in Table 2, 3 and 4, GLT did not affect the activity of liver enzymes, glucose levels, kidney function or the lipid metabolism. In addition, H&E staining of liver, kidney and spleen did not show any pathological changes in these organs (not shown).

**Table 2:** Effect of GLT on the liver function and serum glucose.

Treatment (mg/kg b. wt.)	Albumin (g/dL)	Bilirubin Total (mg/dL)	ALP (IU/L)	ALT (IU/L)	AST (IU/L)	Glucose (mg/dL)
Control	1.3±0.2	0.44±0.15	37±14	54±21	115±37	142±54
GLT 125	1.4±0.2	0.42±0.08	48±19	170±175	161±116	145±34
GLT 250	1.3±0.2	0.30±0.07	38±17	78±30	105±29	119±30
GLT 500	1.3±0.1	0.37±0.10	53±16	151±262	123±121	144±32

Values are Mean ± S.D. (n = 6), ALP, alkaline phosphatase; ALT, alanine transaminase; AST, aspartate transaminase. No significant difference from the control group.

**Table 3:** Effect of GLT on the renal function.

Treatment (mg/kg b. wt.)	Creatinine (mg/dL)	BUN (mg/dL)	Sodium (mg/dL)	Potassium (mmol/L)	Chloride (mmol/L)
Control	0.31±0.05	26.0±3.0	154±0.8	8.3±1.1	114±1.6
GLT 125	0.31±0.02	26.2±2.9	154±1.0	8.4±0.8	112±1.6
GLT 250	0.27±0.05	26.7±7.7	155±1.6	8.2±0.9	113±1.8
GLT 500	0.29±0.03	23.5±2.6	154±0.5	8.5±1.2	113±1.5

Values are Mean ± S.D. (n = 6), BUN, blood urea nitrogen. No significant difference from the control group.

**Table 4:** Effect of GLT on the lipid metabolism.

Treatment (mg/kg b. w.)	Cholesterol (mg/dL)	Triglyceride (mg/dL)	HDL (mg/dL)
**Control**	90±26	164±58	89±21
**GLT 125**	84±4	225±71	78±9
**GLT 250**	97±25	174±90	91±28
**GLT 500**	94±21	245±44	93±19

Values are Mean ± S.D. (n = 6), HDL, high-density lipoprotein. No significant difference from the control group.

**Figure 3:**
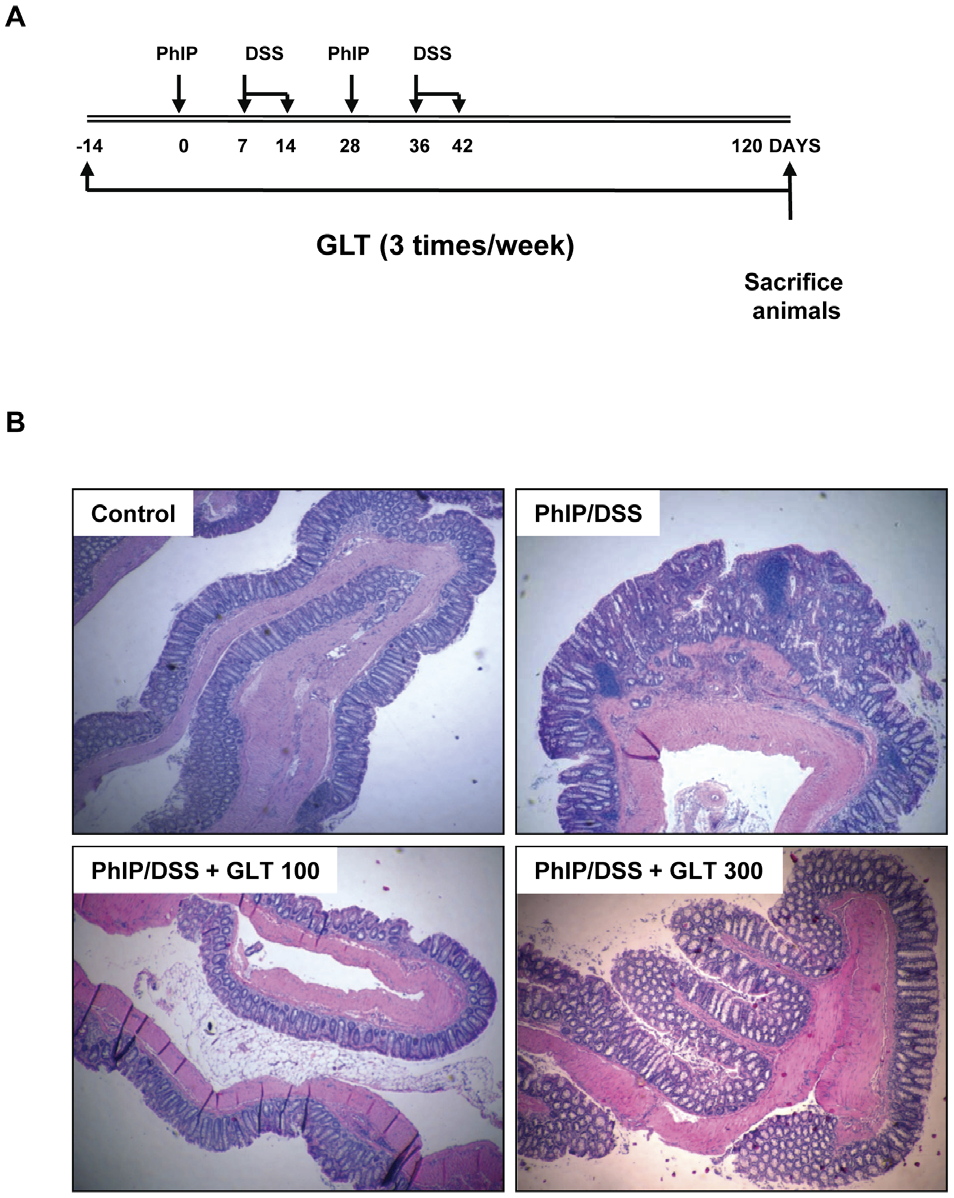
GLT prevents PhIP/DSS induced formation of colon tumors. (A) Schematic of the preventive experimental protocol. The details of the treatment are described in *Materials and Methods*. (B) H&E staining of representative samples from animal experiments.

To evaluate if GLT possesses preventative activity against colon carcinogenesis and inflammation, we started an oral application of GLT (0, 100, 300 mg/kg of body weight) 2 weeks before the initiation of colon carcinogenesis (Fig. 3A). Although some animals died during the time of experiment (2 in the control group, 4 in the PhIP/DSS group, 6 in the PhIP/DSS+100 GLT group, and 3 in the PhIP/DSS+300 GLT group) this mortality is not associated with GLT as demonstrated by the same increase in body weight in all experimental groups (not shown) and the low mortality in the high dose GLT group, further confirming that GLT is not toxic As in our pilot study (Table 1), PhIP/DSS increased tumor formation which was prevented by the oral application of GLT before the tumor initiation (Fig. 3B, Table 5). Moreover, both low (100 mg GLT) and high (300 mg GLT) doses significantly prevented tumor incidence and tumor multiplicity (Table 5). In addition, the amount of tumors was significantly increased in the distal part of the colon, and GLT prevented formation of these tumors (Table 6). As expected, pretreatment with GLT, before the initiation of inflammation with DSS, significantly suppressed colon inflammation as demonstrated by the prevention of the shortening of colon length (Table 5).

**Table 5:** Chemopreventive effect of GLT on the PhIP/DSS induced colon carcinogenesis and inflammation.

Treatment	Tumor incidence (animals with tumors)	Tumor multiplicity (tumors per animal)	Neoplastic index	Colon length (cm)
**Control**	0/12 (0%)	0	0 (0,0)	14.0±1.5
**PhIP/DSS**	26/26 (100%)^a^	4.5±2.2^a^	2 (0,4)^a^	12.1±1.5^a^
**PhIP/DSS+100 GLT**	12/24 (50%)^b^	0.6±0.7^b^	1 (0,4)	13.2±1.2^b^
**PhIP/DSS+300 GLT**	7/27 (26%)^b^	0.3±0.6^b^	0 (0,4)^b^	13.5±1.1^b^

Tumor incidence are summarized using percentage of animals with tumors and compared across groups using Fisher's exact test and the Bonferonni correction for multiple comparisons: ^a^ p<0.001 PhIP/DSS vs control, ^b^ p<0.001 PhIP/DSS+100 GLT vs PhIP/DSS, PhIP/DSS+300 GLT vs PhIP/DSS.

Tumor multiplicity are summarized using mean ± SD and compared across all group using ANOVA on ranks and the Dunn's method for the multiple comparisons: ^a^ p<0.05 PhIP/DSS vs control, ^b^ p<0.05 PhIP/DSS+100 GLT vs PhIP/DSS, PhIP/DSS+300 GLT vs PhIP/DSS.

Neoplastic index is summarized using median (min, max) and compared across groups using the Kruskal-Wallis test. Comparisons of each group to control performed using Mann-Whitney U tests with significance levels adjusted using the Bonferroni correction: ^a^ p<0.001 control vs PhIP/DSS; ^b^ p<0.001 PhIP/DSS vs PhIP/DSS+300 GLT.

Data for colon length summarized using mean ± SD and compared across all group using ANOVA and Dunnett's post hoc test: ^a^p<0.05 control vs PhIP/DSS; ^b^p<0.05 PhIP/DSS vs PhIP/DSS+100 GLT, PhIP/DSS vs PhIP/DSS+300 GLT.

**Table 6:** Tumor localization.

	Tumor localization in colon		
Treatment	proximal	middle	distal
**Control**	0	0	0
**PhIP/DSS**	0.2±0.7	0.7±1.0	3.6±1.6^a^^,^ b ^,^ c
**PhIP DSS+100 GLT**	0	0.2±0.4	0.4±0.6
**PhIP/DSS+300 GLT**	0	0.0±0.2	0.3±0.6

Tumor localization are summarized using mean ± SD and compared across all group using ANOVA on ranks and the Tukey Test (PhIP/DSS group) and the Dunn's method (distal tumors) for the multiple comparisons:

a p<0.05 distal vs proximal, distal vs middle;

b p<0.05 PhIP/DSS vs control;

c p<0.05 PhIP/DSS vs PhIP/DSS+100 GLT, PhIP/DSS vs PhIP/DSS+300 GLT.

### GLT inhibits PhIP/DSS-dependent expression of cyclin D1 and COX-2 in colon tissue

**Figure 4:**
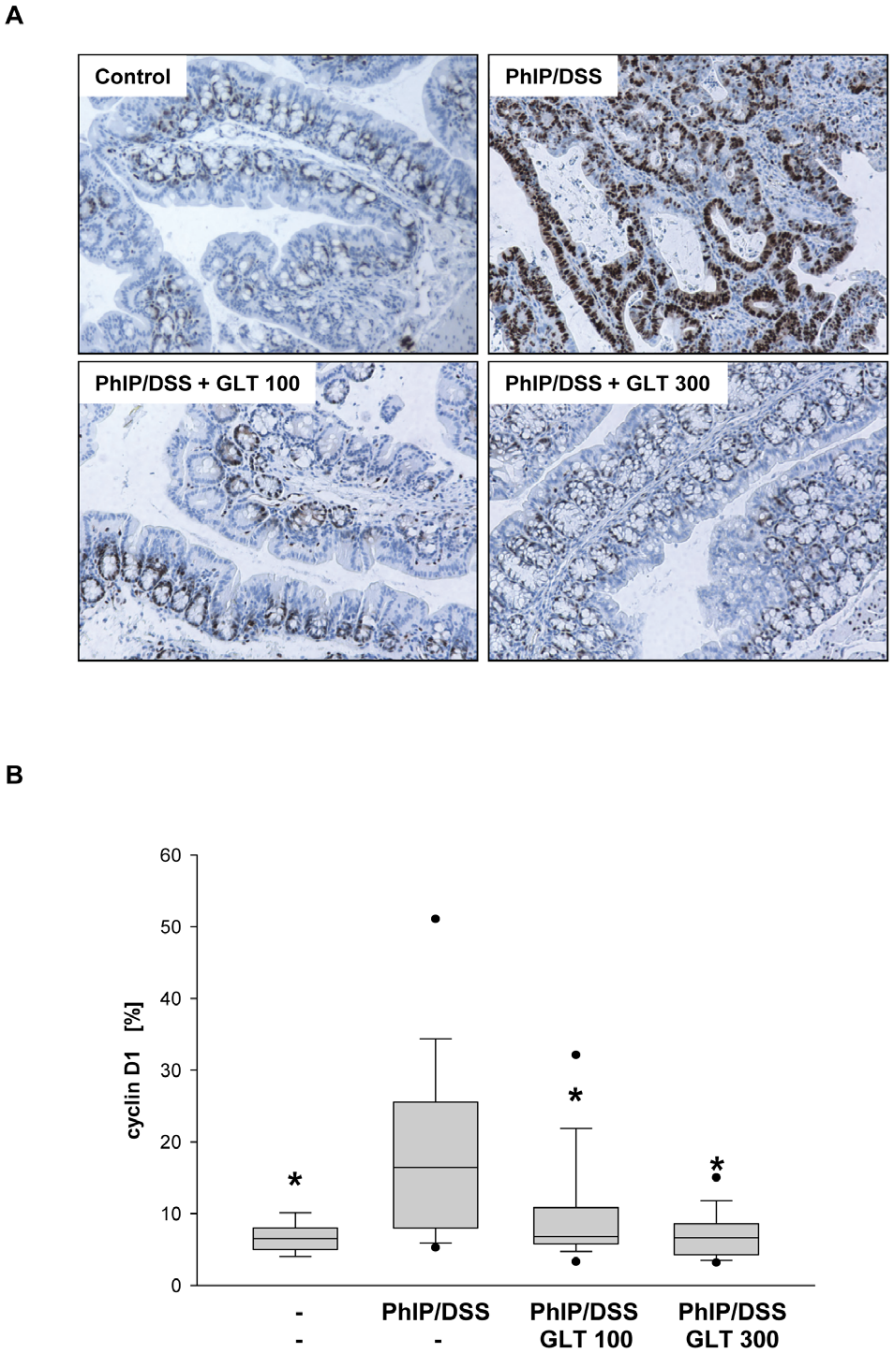
GLT down-regulates expression of cyclin D1 in colon tissue. (A) Immunohistochemistry and (B) quantification of cyclin D1 positive cells were performed as described in *Materials and Methods*. Box plots represent 5^th^/10^th^ percentiles, horizontal bars represent median values, and whiskers indicate minimum to maximum values. Significant differences (*p<0.05) were observed among PhIP/DSS vs. control, PhIP/DSS vs PhIP/DSS+GLT 100, and PhIP/DSS vs. PhIP/DSS+GLT 100.

Overexpression of cell cycle regulatory protein, cyclin D1, was detected in patients with adenomatous polyps and adenocarcinomas demonstrating its importance in colon carcinogenesis [28]. Moreover, PhIP induced expression of cyclin D1 in rat mammary gland carcinomas [29]. Therefore, we evaluated whether the PhIP/DSS will increase the expression of cyclin D1 in colon tissue and whether GLT can reverse this effect. As seen in Figure 4, cyclin D1 expression was markedly increased in mice which received PhIP and DSS, whereas in PhIP/DSS mice treated with 100 and 300 mg GLT/kg the expression of cyclin D1 was decreased.

**Figure 5:**
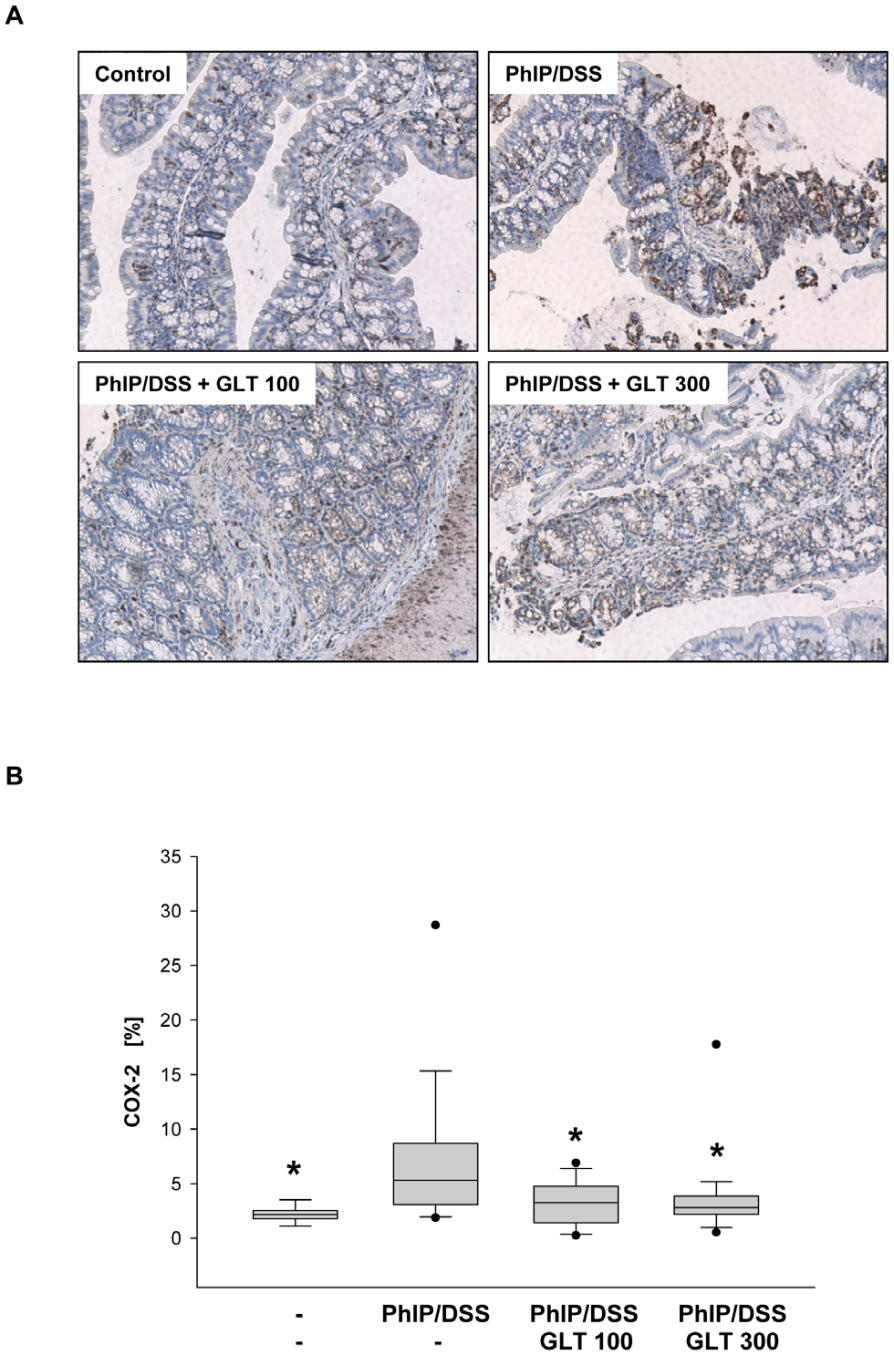
GLT down-regulates expression of COX-2 in colon tissue. (A) Immunohistochemistry and (B) quantification of COX-2 positive cells were performed as described in *Materials and Methods*. Box plots represent 5^th^/10^th^ percentiles, horizontal bars represent median values, and whiskers indicate minimum to maximum values. Significant differences (*p<0.05) were observed among PhIP/DSS vs. control, PhIP/DSS vs PhIP/DSS+GLT 100, and PhIP/DSS vs. PhIP/DSS+GLT 100.

Up-regulation of expression of COX-2, an enzyme converting arachidonic acid to prostaglandins, has been associated with human intestinal inflammation and colorectal cancer [30]. In contrast to the cardiovascular and gastrointestinal effects of COX inhibitors, their use for cancer prevention and treatment of selected patients was recently reevaluated [30]. In agreement with Tanaka et al. [18], PhIP/DSS treatment significantly increased the amount of COX-2 positive cells, whereas GLT treatment (100 and 300 mg GLT/kg) decreased the expression of COX-2 (Fig. 5).

### GLT affects activity of transcription regulators of metabolic enzymes *in vitro* and modulates expression of CYP1A2, CYP3A4 and CYP3A1 *in vivo*

**Figure 6.GLT:**
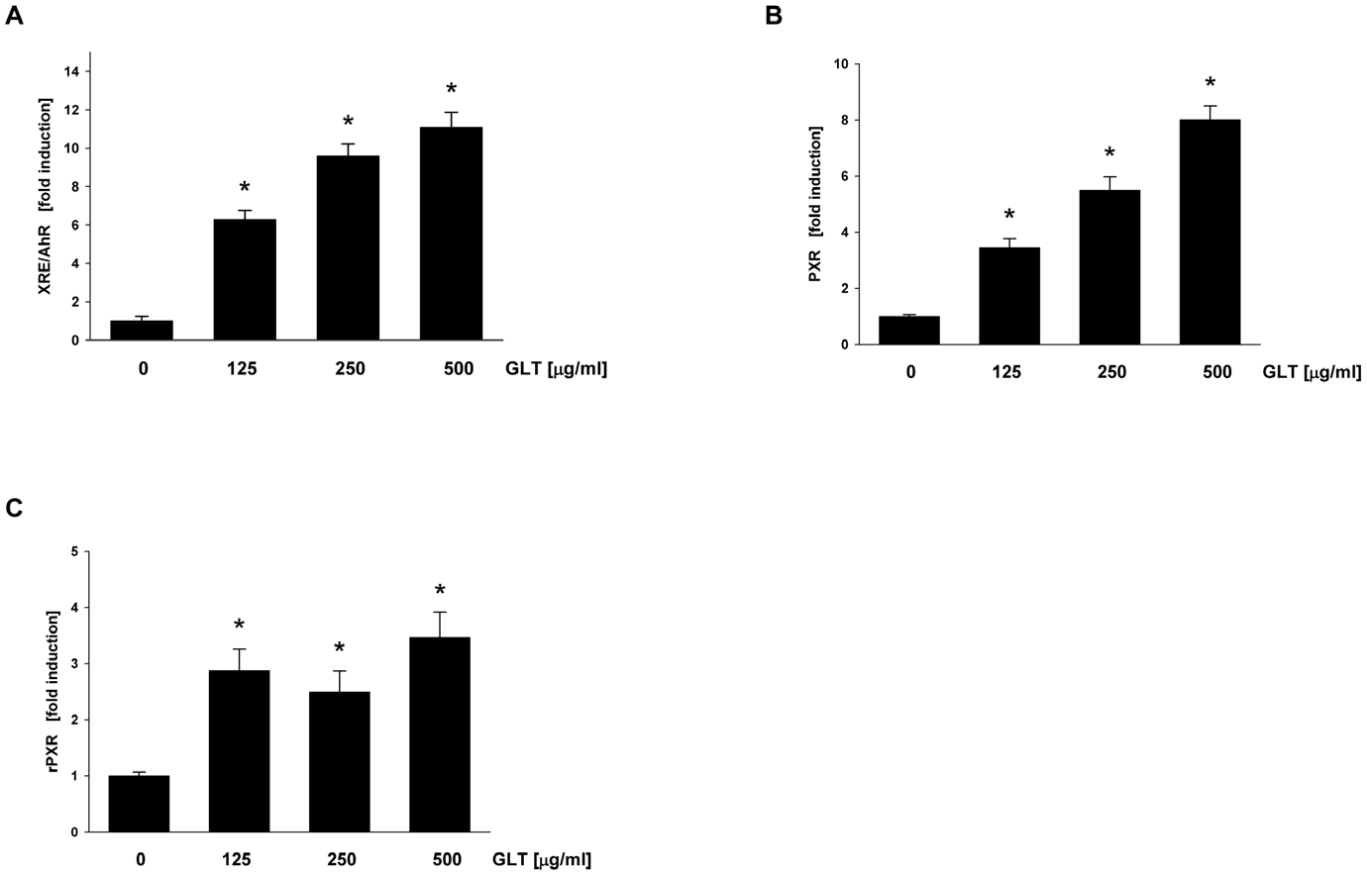
GLT activates XRE/AhR, PXR and rPXR. *in vitro*. Induction of (A) XRE/AhR, (B) PXR and (C) rPXR was evaluated as described in *Materials and Methods*. Results are means ± SD (n = 4), *p<0.05 by ANOVA.

Metabolism of PhIP is mediated by the cytochrome P450 1A2 (CYP1A2) enzyme, leading to the activation or detoxification of PhIP [31]. To determine if GLT has the potential to activate transcription of metabolic enzymes, we have utilized stable cells lines, with reporter luciferase gene constructs containing following promoter regions: (1) XRE (xenobiotic DNA response element) and AhR (Aryl hydrocarbon receptor) coding sequence (human CYP1A2 gene promoter), (2) PXR (human pregnane X receptor DNA response element) coding sequence (human CYP3A4 gene promoter region), and (3) rPXR (rat pregnane X receptor) coding sequence (rat CYP3A1 gene promoter), as described in *Materials and Methods*. The use of the cells containing PXR and rPXR allows for the comparison of induction through human and rat PXR as their response is species specific [32]. The cells containing XRE/AhR, PXR and rPXR were treated with GLT (0–500 µg/ml) for 24 hours, and then assayed for viability and luciferase activity. As seen in Figure 6, GLT significantly activated human XRE/AhR (Fig. 6A) as well as human PXR (Fig. 6B) and rat rPXR (Fig. 6C), without any effect on cell viability. GLT bound rodent PXR with a higher affinity (calculated half maximal effective concentration, EC_50_ of 65.8 µg/ml) than human PXR (calculated EC_50_ of 731.7 µg/ml, which is outside the highest concentration tested), however, GLT was a more potent inducer of human PXR that rodent PXR (eight fold maximum induction at 500 µg/ml vs. three fold maximum induction at 125 µg/ml). GLT had a slightly higher affinity for Ah receptor (calculated EC_50_ of 187.1 µg/ml, eleven fold maximum induction at 500 µg/ml).

**Figure 7:**
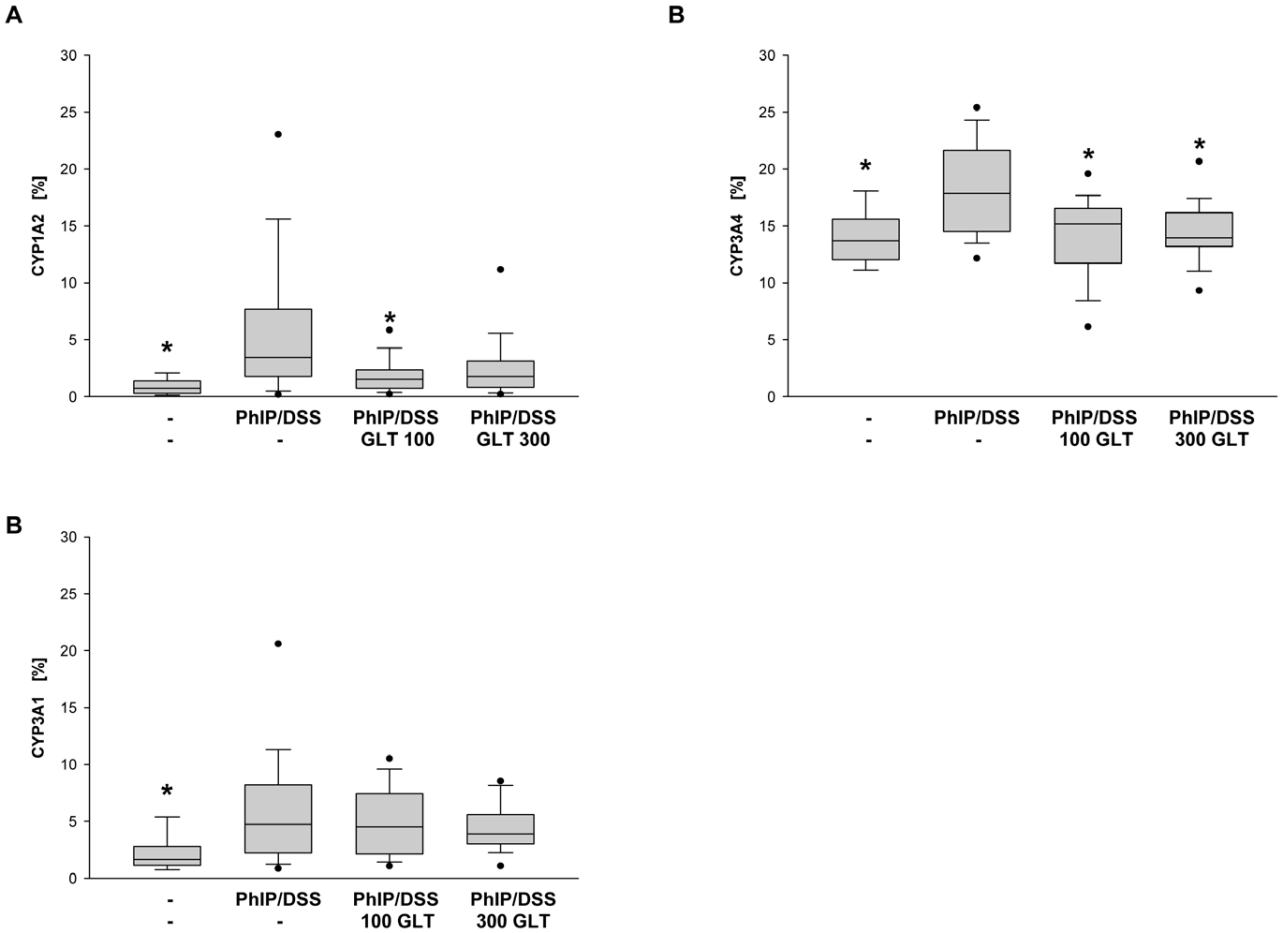
Effect of GLT on PhIP/DSS-induced expression of CYP1A2, CYP3A4 and CYP3A1 in colon tissue. Quantification of (A) CY1A2, (B) CYP3A4, and (C) CYP 3A1 positive cells as described in *Materials and Methods*. Box plots represent 5^th^/10^th^ percentiles, horizontal bars represent median values, and whiskers indicate minimum to maximum values. Significant differences (*p<0.05) were observed among (A) CYP1A2: PhIP/DSS vs. control, PhIP/DSS vs PhIP/DSS+GLT 100 (B) CYP3A4: PhIP/DSS vs. control, PhIP/DSS vs PhIP/DSS+GLT 100, and PhIP/DSS vs. PhIP/DSS+GLT 100, and (C) CYP3A1: PhIP/DSS vs. control.

In addition, we evaluated if GLT treatment affected expression of CYP1A2, CYP3A4 and CYP3A1 *in vivo*, in colonic tissues in mice exposed to PhIP/DSS and GLT treatment. Immunohistochemical analysis demonstrated that PhIP/DSS treatment markedly induced expression of CYP1A2, CYP3A4 and CYP3A1, whereas GLT treatment at 100 and 300 mg/kg decreased expression of CYP1A2 and CYP3A4 to the normal levels (Fig. 7A, B), whereas CYP3A1 expression was not markedly affected (Fig. 7C).

## Discussion

Regardless of the significant global consumption of dietary or medicinal mushrooms, only four epidemiological studies suggested chemopreventive effects of mushrooms against gastric, gastrointestinal and breast cancer, respectively [7]–[10]. In the present study, we evaluated anti-cancer and anti-inflammatory activities of a triterpene extract from medicinal mushroom *Ganoderma lucidum* (GLT) in an animal model of the food-borne carcinogen (PhIP) and inflammation (DSS) induced colon carcinogenesis. Our data clearly supports our hypothesis that GLT prevents and suppresses both colon carcinogenesis and colon inflammation in ICR mice exposed to PhIP and DSS. Previous study using uncharacterized water soluble extract from cultured medium of *G. lucidum* mycelia (MAK), demonstrated that MAK in the diet reduced the size but not the amount of colon tumors induced by N,N′-dimethylhydrazine (DMH) in ICR mice [16]. Therefore, our study is the first to demonstrate that chemically characterized extract from mushroom *G. lucidum*, GLT, suppresses the number of colon tumors induced by the food-borne carcinogen and inflammation.

As recently demonstrated, an induction of preneoplastic lesions in rat colon by PhIP was not preceded or accompanied by an inflammatory process [33], whereas the incidence and multiplicity of dysplastic lesions were promoted by the addition of DSS [18]. Indeed, here we demonstrate that focal hyperplasia induced by PhIP was further accelerated by DSS. Most importantly, GLT suppressed colonic hyperplasia induced by the combination of PhIP and DSS in a dose response manner, further confirming the role of GLT in the prevention of colon carcinogenesis. The inhibition of hyperplasia was also confirmed by the suppression of the proliferative marker Ki-67 in colonic tissue in mice treated with GLT. In addition, we have found that although PhIP induced ACF, DSS-initiated inflammation further increased the amount of ACF in mice treated with PhIP and DSS. In agreement with the study using an extract from *G. lucidum* mycelia MAK [16], GLT inhibited ACF formation in ICR mice in a dose-responsive manner.

Increased mortality, in our pilot and preventive studies, could be caused by the toxicity of DSS and by the additional effect of GLT. Because DSS (inflammation) itself induces mice mortality [34], and all death animals (but 1 in the pilot and 2 in the preventive control groups) were exposed to DSS, inflammation could be responsible for this increased mortality. Moreover, COX-2 knock-out mice, specifically in myeloid and endothelial cells but not epithelial cells, are more sensitive to DSS [35], [36], and GLT treatment markedly decreased expression of COX-2 in mice treated with DSS and PhIP in our study. Therefore, it is possible that GLT inhibition of COX-2 further sensitize mice to DSS, resulting in the increased mortality in the PhIP/DSS and PhIP/DSS+GLT treatment groups. In addition, as in our both studies, we have observed an increased aggressive behavior in the gavaged mice.

Here, we also demonstrate anti-inflammatory effects of GLT in the PhIP/DSS-model of colon carcinogenesis. DSS-induced inflammation/colitis is associated with the shortening of the large intestine in DSS treated animals [25], and we observed the same effect in the PhIP/DSS treated animals. However, GLT prevented the shortening of colon in PhIP/DSS treated animals to the colon length comparable to control animals, suggesting anti-inflammatory effect of GLT in the PhIP/DSS model of colon carcinogenesis.

The tumor microenvironment contains stromal cells such as fibroblasts, endothelial cells, and macrophages. An association of chronic inflammation, which involves macrophages, with cancer initiation and promotion and the reduction of cancer risk by treatment with anti-inflammatory drugs was recently described [37]. As mentioned above, increased infiltration of macrophages was previously detected in the DSS-dependent colon inflammation [26]. Our data demonstrate that PhIP/DSS induce the amount of infiltrating macrophages whereas GLT treatment significantly reduced their amount. Therefore, in addition to the inhibition of colon carcinogenesis, our data suggest that GLT exerts its effect through the inhibition of inflammation and by the depletion of infiltrating macrophages from colon tissue. Moreover, we have recently demonstrated direct anti-inflammatory activity of GLT in LPS-stimulated macrophages *in vitro* and in LPS-challenged mice *in vivo* [38]. Hence, our data are in agreement with previous study by Loddenkemper *et al.* demonstrating that ursodeoxycholic acid, another triterpene with anti-inflammatory properties, prevented colitis-associated carcinogenesis in mice [33].

The overexpression of cyclin D1 and COX-2 in colon tissues in animals treated with PhIP/DSS was markedly reduced by the GLT treatment. We have previously demonstrated that an extract of mushroom *G. lucidum*, which contains 6% of GLT, suppressed cyclin D1 expression and NF-κB activity in human breast cancer cells [39]. Moreover, GLT down-regulated expression of COX-2 and suppressed activation of AP-1 and NF-κB in stimulated macrophages [38]. Therefore, it is plausible to hypothesize that GLT down-regulates the expression of cyclin D-1 and COX-2 *in vivo* by inhibiting AP-1 and NF-κB activity. Indeed, recent study demonstrated that euphol, a natural triterpene from the *Euphorbia tirucalli* plant, inhibits NF-κB activity in colon tissue from mice the DSS-induced colitis [40].

As previously demonstrated, polysaccharides isolated from *G. lucidum*, induced total P450 levels in rat liver, whereas suppressed the activity of CYP1A2, CYP3A and CYP2E1 in hepatic microsomes [41]. Our results indicate that GLT is able to induce XRE/AhR and PXR which regulate expression of CYP1A2 and CYP3A, respectively. Because CYP1A2 is involved in the metabolism of PhIP [30], it is possible that GLT-induced increase in the expression of CYP1A2, could contribute to the elimination of this carcinogen. Moreover, PXR can be also responsible for the GLT-induced down-regulation of expression of cyclin D1. Since the ligand bound PXR heterodimerizes with the retinoid X receptor (RXR) and this interaction is required for an induction of the gene expression [42], the depletion of the RXR resulted in a decrease in cyclin D1 expression [43]. Therefore, the decrease in cyclin D1 expression by GLT treatment could be caused by the interaction between PXR, GLT and RXR. Moreover, the expression of PXR regulated genes are decreased in inflammatory bowel disease [44], suggesting that GLT induced PXR could account of decreased inflammation seen in our study. It is not unusual that GLT is able to activate both PXR and AhR mediated gene expression since Omeprazole (a dyspeptic drug) is able to activate both PXR and AhR (J. Lamb, personal communication), and a “cross-talk” between PXR and AhR has been described [44]. The PhIP/DSS-induced expression of CYP1A2 in mice is an agreement with previous study demonstrating increased expression of this enzyme by PhIP in rat [45]. Although the metabolism of PhIP by CYP1A2 differs between human and rodents, the inhibition of a PhIP/DSS-dependent expression of CYP1A2 by GLT can reduce the amount of carcinogenic PhIP metabolite N-hydroxy-PhIP in colon tissue [31]. Since, Curcuma drugs suppressed activity and expression of CYP3A4 in intestinal epithelial cells [46], our observation that GLT down-regulates PhiP/DSS-dependent expression of CYP3A4 *in vivo*, further confirms cancer preventative activities of GLT.

In our studies we used GLT concentrations up to 500 mg/kg of body weight 3 times per week, which would correspond 90–120 g of GLT for 60–80 kg per person per week (12.9–17.1 g/day). Since the amount of biologically active mushroom triterpenes (GLT) depends on the source and extraction procedures, the consumption of 1–2 servings (100–200 grams) of fresh or dried mushrooms could be sufficient to reach these concentrations. In addition, daily intake of fresh (≥10 g) or dried (≥4 g) mushrooms significantly decreased the risk of breast cancer [9]. In summary, our data demonstrate, for the first time, that GLT prevented and inhibited colon carcinogenesis in mice which was induced by the food-borne carcinogen and inflammation. Moreover, GLT also suppressed colon inflammation, reduced the amount of infiltrating macrophages and down-regulated expression of cyclin D1 and COX-2, and suppressed PhIP/DSS-induced expression of CYP1A and CYP3A in colon tissue. Taken together, GLT could be considered as potential natural agent for the prevention and suppression of colitis-associated colon carcinogenesis.

## Materials and Methods

### *Ganoderma lucidum* extract and reagents

*Ganoderma lucidum* triterpene extract (GLT) was obtained from Pharmanex (Provo, UT, USA, batch number: 050607, Shanghai R&D, Pharmanex). GLT contains a mixture of lanostanoid triterpenes which we previously characterized and quantified: ganoderic acid A, F, H, Mh, S1, ganosporeric acid, lucidenic acid B, D, D1, E1, H, L, and methyl lucidenate G [47]. GLT was dissolved in drinking water at the concentration 10 and 50 mg/ml for animal experiments and in DMSO (Sigma, St. Louis, MO, USA) at the concentration 40 mg/ml for cell culture experiments and stored at 4°C, respectively. PhIP was purchased from Toronto Research Chemicals, Inc. (Toronto, Ontario, Canada). DSS was obtained from MP Biomedicals (Solon, OH, USA).

### Animal experiments

Male Crj: CD-1 (ICR) mice (Harlan, Indianapolis, USA) 5 weeks old were maintained at Methodist Research Institute Animal Facility according to the Institutional Animal Care Guidelines. All animals were housed in plastic cages (4 mice/cage) with free access to drinking water and a pellet diet (LabDiet 5001, Southern Agriculture, Tulsa, OK, USA), under controlled conditions of humidity (50±10%), light (12/12 h light/dark cycle) and temperature (23±2°C). After 7 days quarantine, they were randomized by body weight into control and experimental groups. To establish a food-borne- and inflammation- model of colon carcinogenesis, ICR mice (10 animals/group) received an intra-gastric dose of PhIP (100 mg/kg of body weight) [day 0 and 28], and 1 week after the PhIP applications, they received 2% (w/v) DSS in drinking water for 7 days [days 7–14 and 36–42]. The control groups received water or PhIP (100 mg/kg of body weight) [day 0 and 28] or DSS in drinking water for 7 days [days 7–14 and 36–42], respectively. The experimental groups were gavaged with GLT dissolved in drinking water (0, 100 mg, 500 mg/kg of body weight) three times per week starting at the day 1 until the end of the experiment (Fig. 1A). In the prevention study, ICR mice were gavaged with corn oil (control group, n = 14; PhIP/DSS group, n = 30) or GLT (PhIP/DSS+100 mg GLT, n = 30; and PhIP/DSS+300 mg/kg GLT, n = 30; of body weight in corn oil) for 2 weeks before the first application of PhIP as described above (Fig. 3A). The mice were sacrificed at day 120 and colon tissue was collected. The large bowels were flushed with saline and excised; the length was measured (from ileocecal junction to the anal verge) and cut open longitudinally along the main axis, and then washed with saline. Macroscopic inspection was carried out and the large bowels were then cut and fixed in 10% buffered formalin for 24 hours followed by tissue processing overnight, and then embedded in paraffin. Five-micrometer sections were stained for routine H&E for the histological analysis.

Toxicity of GLT was evaluated in male ICR mice with GLT (0, 125, 250 and 500 mg/kg) via the p.o. route of administration, and dissolved in corn oil (n = 6). 7 days after the administration initiation of the dosing (5×, daily dosing), a full necropsy was performed and liver, kidney and spleen collected for the histological analysis as described above (H&E staining). Blood was collected and levels of albumin, bilirubin, ALP, ALT, AST, glucose, creatinine, BUN, sodium, potassium, chloride, cholesterol, triglyceride and HDL determined at the IU Health Pathology Laboratory (Indianapolis, Indiana, USA)

The protocols for animal experiments were approved by the Animal Research Committee at the Methodist Hospital (protocol number 2006-22, 2012-07) according to the NIH guidelines for the Care and Use of Laboratory Animals.

### Immunohistochemistry

Immunohistochemistry for Ki-67, F4/80, cyclin D1 and COX-2 (Santa Cruz Biotechnology Inc., Santa Cruz, CA, USA), CYP1A2 (Abcam Inc., Cambridge, MA, USA), CYP 3A1 (Biorbyt Ltd., Cambridge, UK), CYP3A4 (Proteintech Group Inc., Chicago, IL, USA) was performed on 5-µm-thick paraffin-embedded sections from the colons of mice. For each case, negative controls were performed on serial sections. For the Ki-67 positive nuclei, (focal hyperplasia/adenoma/tumor areas) nuclei were measured in one colon section per mouse and expressed as the number of positive cells per 16× power field (160×) in the colon cross-sectional area. For the immunohistochemical quantification, three randomly selected images (16× power fields) each (total area, 7.3 mm^2^) were analyzed in each animal per group. The macrophage infiltration in the colonic tissues was evaluated by the immunostaining detection of macrophage/monocyte marker (F4/80) monoclonal primary antibody and the number of macrophages were counted in three 16× fields and averaged. Cyclin D1,COX-2, CYP1A2, CYP3A1 and CYP3A4 expression were evaluated in colon tissues in all survived animals (one slide per animal) from each group in the prevention study (control, n = 12; PhIP/DSS, n = 26; PhIP/DSS+100 GLT, n = 24; PhIP/DSS+300 GLT, n = 27) by ImageJ [48]. Intensity and localization of immunoreactivities against all primary antibodies used were examined on all sections using a light microscope (Leica DMR type 020-525-024 fluorescence microscope, Wetzlar, Germany).

### Scoring of Aberrant Crypts

For the counting of aberrant crypts unstained slides with paraffin embedded tissues were selected as described [49]. Total three slides corresponding to three different animals (one slide per animal) from each group were used. First the slides were treated with xylene and different concentration of ethanol for different timings to remove paraffin from tissues. After removal of paraffin, tissues were stained with 0.2% methylene blue for 3 minutes in distilled water, and viewed under a light microscope to determine aberrant crypts (AC). The AC were distinguished from surrounding non-aberrant crypts by their increased size, thickened epithelial cell lining, and enlarged pericryptal area relative to surrounding normal crypts. 10 different foci (fields) were selected from each slide to count the aberrant crypts. The AC from all 10 foci were added together for each slide. Lastly, average number of aberrant crypts from all 30 foci was determined together.

### Neoplastic Index

Histological examination was performed on paraffin-embedded sections after hematoxylin and eosin (H&E) staining for the histological alterations, such as mucosal ulceration, dysplasia, and colonic neoplasms according to the description by Ward [50]. Neoplastic index was based on the evaluation of neoplastic lesions which were scored in the following manner: mildly/poorly developed = 1, moderately developed = 2, highly/well developed = 3, very highly/well developed = 4, as described by Nakagama [51]. After complete scoring the average score was determined as neoplastic index.

### *In vitro* Assays

1A2DRE (human CYP1A2 XRE), DPX2 (human CYP3A4 PXR), and RPXR (rat CYP3A1) cells, obtained from Puracyp Inc. (Carlsbad, CA, USA), were exposed to GLT in a 96 well plate for 24 hours. Viability was determined by MultiTox (Promega, Madison, WI, USA) at 405_ex_/530_em_ nm and induction was determined by luminescent activity using BrightGlo (Promega), in a BioTek Synergy2 plate reader. EC_50s_ and calculated maximum induction were determined using Sigma Plot software.

### Statistical analysis

Tumor incidences were summarized using percentage of animals with tumors and compared across groups using Fisher's exact test and the Bonferonni correction for multiple comparisons. Tumor multiplicity were summarized using mean ± SD and compared across all group using Kruskal-Wallis one way analysis of variance on ranks and the Dunn's method for the multiple comparisons. Neoplasia data were summarized using median (min, max) and compared across groups using the Kruskal-Wallis test. Comparisons of each group to control was performed using Mann-Whitney U tests with significance levels adjusted using the Bonferroni correction. Colon length data were summarized using mean (SD) and compared across all groups using ANOVA and Dunnett's post hoc test. All other data were summarized using mean ± SD and compared across groups using ANOVA.

## References

[1] Sugimura T (2000) Nutrition and dietary carcinogens. Carcinogenesis 21: 387–395. 10688859 10.1093/carcin/21.3.387

[2] Sinha R, Peters U, Cross AJ, Kulldorff M, Weissfeld JL, et al (2005) Meat, meat cooking methods and preservation, and risk for colorectal adenoma. Cancer Res 65: 8034–8041. 16140978 10.1158/0008-5472.CAN-04-3429

[3] Rohrmann S, Hermann S, Linseisen J (2009) Heterocyclic aromatic amine intake increases colorectal adenoma risk: findings from a prospective European cohort study. Am J Clin Nutr 89: 1418–1424. 19261727 10.3945/ajcn.2008.26658

[4] Hussain SP, Harris CC (2007) Inflammation and cancer: an ancient link with novel potentials. Int J Cancer 121: 2373–2380. 17893866 10.1002/ijc.23173

[5] van Hogezand RA, Eichhorn RF, Choudry A, Veenendaal RA, Lamers CBHW (2002) Malignancies in inflammatory bowel disease: fact or fiction? Scand J Gastroenter - Suppl: 48–53. 10.1080/003655202320621454 12408504

[6] World Cancer Research Fund/American Institute for Cancer Research. Food, Nutrition, Physical Activity, and the Prevention of Cancer: a Global Perspective. Washington, DC: AICR. 2007.

[7] Kim HJ, Chang WK, Kim MK, Lee SS, Choi BY (2002) Dietary factors and gastric cancer in Korea: a case-control study. Int J Cancer 97: 531–535. 11802218 10.1002/ijc.10111

[8] Hara M, Hanaoka T, Kobayashi M, Otani T, Adachi HY, et al (2003) Cruciferous vegetables, mushrooms, and gastrointestinal cancer risks in a multicenter, hospital-based case-control study in Japan. Nutr Cancer 46: 138–147. 14690789 10.1207/S15327914NC4602_06

[9] Zhang M, Huang J, Xie X, Holman CDAJ (2009) Dietary intakes of mushrooms and green tea combine to reduce the risk of breast cancer in Chinese women. Int J Cancer 124: 1404–1408. 19048616 10.1002/ijc.24047

[10] Shin A, Kim J, Lim SY, Kim G, Sung MK, et al (2010) Dietary mushroom intake and the risk of breast cancer based on hormone receptor status. Nutr Cancer 62: 476–483. 20432168 10.1080/01635580903441212

[11] Borchers AT, Krishnamurthy A, Keen CL, Meyers FJ, Gershwin ME (2008) The immunobiology of mushrooms. Exp Biol Medicine 233: 259–276. 10.3181/0708-MR-227 18296732

[12] Mattila P, Suonpaa K, Piironen V (2000) Functional properties of edible mushrooms. Nutrition 16: 694–696. 10906601 10.1016/s0899-9007(00)00341-5

[13] Sliva D (2006) *Ganoderma lucidum* in cancer research. Leukemia Res 30: 767–768. 16458355 10.1016/j.leukres.2005.12.015

[14] Kimura Y, Taniguchi M, Baba K (2002) Antitumor and antimetastatic effects on liver of triterpenoid fractions of *Ganoderma lucidum*: mechanism of action and isolation of an active substance. Anticancer Res 22: 3309–3318. 12530080

[15] Weng CJ, Chau CF, Yen GC, Liao JW, Chen DH, et al (2009) Inhibitory effects of *Ganoderma lucidum* on tumorigenesis and metastasis of human hepatoma cells in cells and animal models. J Agric Food Chem 57: 5049–5057. 19422227 10.1021/jf900828k

[16] Lu H, Kyo E, Uesaka T, Katoh O, Watanabe H (2002) Prevention of development of N,N′-dimethylhydrazine-induced colon tumors by a water-soluble extract from cultured medium of *Ganoderma lucidum* (Rei-shi) mycelia in male ICR mice. Int J Mol Med 9: 113–117. 11786919

[17] Lu H, Uesaka T, Katoh O, Kyo E, Watanabe H (2001) Prevention of the development of preneoplastic lesions, aberrant crypt foci, by a water-soluble extract from cultured medium of *Ganoderma lucidum* (Rei-shi) mycelia in male F344 rats. Oncol Rep 8: 1341–1345. 11605062 10.3892/or.8.6.1341

[18] Tanaka T, Suzuki R, Kohno H, Sugie S, Takahashi M, et al (2005) Colonic adenocarcinomas rapidly induced by the combined treatment with 2-amino-1-methyl-6-phenylimidazo[4,5-b]pyridine and dextran sodium sulfate in male ICR mice possess beta-catenin gene mutations and increases immunoreactivity for beta-catenin, cyclooxygenase-2 and inducible nitric oxide synthase. Carcinogenesis 26: 229–238. 15459021 10.1093/carcin/bgh292

[19] Tanaka T (2009) Colorectal carcinogenesis: Review of human and experimental animal studies. J Carcinogenesis 8: 5. 10.4103/1477-3163.49014 PMC2678864 19332896

[20] Gupta AK, Schoen RE (2009) Aberrant crypt foci: are they intermediate endpoints of colon carcinogenesis in humans? Curr Op Gastroenterol 25: 59–65. 10.1097/MOG.0b013e32831db286 19114775

[21] Steffensen IL, Paulsen JE, Eide TJ, Alexander J (1997) 2-Amino-1-methyl-6-phenylimidazo[4,5-b]pyridine increases the numbers of tumors, cystic crypts and aberrant crypt foci in multiple intestinal neoplasia mice. Carcinogenesis 18: 1049–1054. 9163695 10.1093/carcin/18.5.1049

[22] Nishikawa A, Imazawa T, Kuroiwa Y, Kitamura Y, Kanki K, et al (2005) Induction of colon tumors in C57BL/6J mice fed MeIQx, IQ, or PhIP followed by dextran sulfate sodium treatment. Toxicol Sci 84: 243–248. 15635146 10.1093/toxsci/kfi079

[23] Kikuchi Y, Dinjens WN, Bosman FT (1997) Proliferation and apoptosis in proliferative lesions of the colon and rectum. Virchows Arch 431: 111–117. 9293892 10.1007/s004280050076

[24] Nakanishi M, Tazawa H, Tsuchiya N, Sugimura T, Tanaka T, et al (2007) Mouse strain differences in inflammatory responses of colonic mucosa induced by dextran sulfate sodium cause differential susceptibility to PhIP-induced large bowel carcinogenesis. Cancer Sci 98: 1157–1163. 17573895 10.1111/j.1349-7006.2007.00528.x PMC11159423

[25] Okayasu I, Hatakeyama S, Yamada M, Ohkusa T, Inagaki Y, et al (1990) A novel method in the induction of reliable experimental acute and chronic ulcerative colitis in mice. Gastroenterol 98: 694–702. 10.1016/0016-5085(90)90290-h 1688816

[26] Islam MS, Murata T, Fujisawa M, Nagasaka R, Ushio H, et al (2008) Anti-inflammatory effects of phytosteryl ferulates in colitis induced by dextran sulphate sodium in mice. Br J Pharmacol 154: 812–824. 18536734 10.1038/bjp.2008.137 PMC2439859

[27] Smina TP, Mathew J, Janardhanan KK, Devasagayam TP (2011) Antioxidant activity and toxicity profile of total triterpenes isolated from Ganoderma lucidum (Fr.) P. Karst occurring in South India. Environ Toxicol Pharmacol 32: 438–446. 22004964 10.1016/j.etap.2011.08.011

[28] Arber N, Hibshoosh H, Moss SF, Sutter T, Zhang Y, et al (1996) Increased expression of cyclin D1 is an early event in multistage colorectal carcinogenesis. Gastroenterology 110: 669–674. 8608874 10.1053/gast.1996.v110.pm8608874

[29] Shan L, He M, Yu M, Qiu C, Lee NH, et al (2002) cDNA microarray profiling of rat mammary gland carcinomas induced by 2-amino-1-methyl-6-phenylimidazo[4,5- b]pyridine and 7,12- dimethylbenz[a]anthracene. Carcinogenesis 23: 1561–1568. 12376462 10.1093/carcin/23.10.1561

[30] Wang D, Dubois RN (2010) The role of COX-2 in intestinal inflammation and colorectal cancer.. Oncogene 29: 781–788. 19946329 10.1038/onc.2009.421 PMC3181054

[31] Cheung C, Ma X, Krausz KW, Kimura S, Feigenbaum L, et al (2005) Differential metabolism of 2-amino-1-methyl-6-phenylimidazo[4,5-b]pyridine (PhIP) in mice humanized for CYP1A1 and CYP1A2. Chem Res Toxicol 18: 1471–1478. 16167840 10.1021/tx050136g

[32] Graham MJ, Lake BG (2008) Induction of drug metabolism: species differences and toxicological relevance. Toxicology 254: 184–191. 18824059 10.1016/j.tox.2008.09.002

[33] Kuhnel D, Taugner F, Scholtka B, Steinberg P (2009) Inflammation does not precede or accompany the induction of preneoplastic lesions in the colon of 2-amino-1-methyl-6-phenylimidazo[4,5-b]pyridine-fed rats. Arch Toxicol 83: 763–768. 19212758 10.1007/s00204-009-0406-2

[34] Mabley JG, Pacher P, Liaudet L, Soriano FG, Haskó G, et al (2003) Inosine reduces inflammation and improves survival in a murine model of colitis. Am J Physiol Gastrointest Liver Physiol 284: G138–144. 12388199 10.1152/ajpgi.00060.2002

[35] Ishikawa TO, Herschman HR (2010) Tumor formation in a mouse model of colitis associated colon cancer does not require COX-1 or COX-2 expression. Carcinogenesis 31: 729–736. 20061361 10.1093/carcin/bgq002 PMC2847091

[36] Ishikawa TO, Oshima M, Herschman HR (2011) Cox-2 deletion in myeloid and endothelial cells, but not in epithelial cells, exacerbates murine colitis. Carcinogenesis 32: 417–426. 21156970 10.1093/carcin/bgq268 PMC3047239

[37] Balkwill F, Charles KA, Mantovani A (2005) Smoldering and polarized inflammation in the initiation and promotion of malignant disease. Cancer Cell 7: 211–217. 15766659 10.1016/j.ccr.2005.02.013

[38] Dudhgaonkar S, Thyagarajan A, Sliva D (2009) Suppression of the inflammatory response by triterpenes isolated from the mushroom *Ganoderma lucidum* . Int Immunopharmacol 9: 1272–1280. 19651243 10.1016/j.intimp.2009.07.011

[39] Jiang J, Slivova V, Harvey K, Valachovicova T, Sliva D (2004) *Ganoderma lucidum* suppresses growth of breast cancer cells through the inhibition of Akt/NF-kappaB signaling. Nutr Cancer 49: 209–216. 15489214 10.1207/s15327914nc4902_13

[40] Dutra RC, Claudino RF, Bento AF, Marcon R, Schmidt EC, et al (2011) Preventive and therapeutic euphol treatment attenuates experimental colitis in mice. PLoS One 6: e27122. 22073270 10.1371/journal.pone.0027122 PMC3206917

[41] Wang X, Zhao X, Li D, Lou YQ, Lin ZB, et al (2007) Effects of *Ganoderma lucidum* polysaccharide on CYP2E1, CYP1A2 and CYP3A activities in BCG-immune hepatic injury in rats. Biol Pharmacol Bull 30: 1702–1706. 10.1248/bpb.30.1702 17827724

[42] Pascussi JM, Gerbal-Chaloin S, Duret C, Daujat-Chavanieu M, Vilarem MJ, et al (2008) The tangle of nuclear receptors that controls xenobiotic metabolism and transport: crosstalk and consequences. Annu Rev Pharmacol Toxicol 48: 1–32. 17608617 10.1146/annurev.pharmtox.47.120505.105349

[43] Yang X, Guo M, Wan YJ (2010) Deregulation of growth factor, circadian clock, and cell cycle signaling in regenerating hepatocyte RXRalpha-deficient mouse livers. Am J Pathol 176: 733–743. 20035057 10.2353/ajpath.2010.090524 PMC2808080

[44] Langmann T, Moehle C, Mauerer R, Scharl M, Liebisch G, et al (2004) Loss of detoxification in inflammatory bowel disease: dysregulation of pregnane X receptor target genes. Gastroenterology 127: 26–40. 15236169 10.1053/j.gastro.2004.04.019

[45] Mori Y, Koide A, Tatematsu K, Sugie S, Mori H (2005) Effects of alpha-naphthyl isothiocyanate and a heterocyclic amine, PhIP, on cytochrome P-450, mutagenic activation of various carcinogens and glucuronidation in rat liver. Mutagenesis 20: 15–22. 15598703 10.1093/mutage/gei001

[46] Hou XL, Takahashi K, Kinoshita N, Qiu F, Tanaka K, et al (2007) Possible inhibitory mechanism of Curcuma drugs on CYP3A4 in 1alpha,25 dihydroxyvitamin D3 treated Caco-2 cells. Int J Pharm 337: 169–177. 17270371 10.1016/j.ijpharm.2006.12.035

[47] Adamec J, Jannash A, Dudhgaonkar S, Jedinak A, Sedlak M, et al (2009) Development of a new method for improved identification and relative quantification of unknown metabolites in complex samples: Determination of a triterpenoid metabolic fingerprint for the in situ characterization of Ganoderma bioactive compounds. J Sep Sci 32: 4052–4058. 19937965 10.1002/jssc.200900496

[48] Vrekoussis T, Chaniotis V, Navrozoglou I, Dousias V, Pavlakis K, et al (2009) Image analysis of breast cancer immunohistochemistry-stained sections using ImageJ: an RGB- based model. Anticancer Res 29: 4995–4998. 20044607

[49] Bobek P, Galbavy S (2001) Effect of pleuran (beta-glucan from *Pleurotus ostreatus*) on the antioxidant status of the organism and on dimethylhydrazine-induced precancerous lesions in rat colon. Br J Biomed Sci 58: 164–168. 11575739

[50] Ward JM (1974) Morphogenesis of chemically induced neoplasms of the colon and small intestine in rats. Lab Invest 30: 505–513. 4363166

[51] Nakagama H, Nakanishi M, Ochiai M (2005) Modeling human colon cancer in rodents using a food-borne carcinogen, PhIP. Cancer Sci 96: 627–636. 16232193 10.1111/j.1349-7006.2005.00107.x PMC11158313

